# The Radcliffe Infirmary, Oxford

**Published:** 1901-02-16

**Authors:** 


### THE RADCLIFFE INFIRMARY, OXFORD.

Much time would be saved when hospital managers meet together to discuss the affairs of their little kingdom if those who wish to give their hobbies a run would take the trouble first of all to discover whether or no they have any backers, for, interesting as it may be to those who have a grievance to air it regularly, it is a waste of time if there is no sort of chance of anything being done. This, of course, is a platitude. Nevertheless, if we may judge by the proceedings at a recent meeting of the governors of the Radcliffe Infirmary, Oxford, at which a long discussion took place on a motion to increase the salary of the chaplain—a motion which was only supported by four votes out of all those present—there are people who regard hospital meetings as occasions on which time is a matter of no moment The whole matter in question had been discussed last year, and the Board had then made an arrangement, which was certainly intended to be a permanent one, according to which, with the object of giving a pension to the former chaplain, a certain reduction was made in the amount of salary to be offered out of the infirmary funds to his successor. We do not propose to comment upon the amount of the pension, nor upon the justifiability of the compromise according to which, for an unknown number of years, the amount of salary of the chaplain was to run on at a lower rate than had formerly been the case; but we do suggest that to bring up the whole question and ask the meeting to take such an extreme measure as to reverse the permanent arrangement made the preceding year, and for this to be done by a misguided cleric who was so out of touch with public opinion and so ignorant of the feelings of his fellow-governors on the subject that he could only find three supporters, was a miserable waste of time. There are certain things in the management of every hospital which by common consent ought not to be brought up for review year after year, and among those things are questions of salary and of pension. Whenever an appointment is made and whenever a pension has to be arranged then the fullest and freest discussion is desirable. But once the matter is decided it ought to stand. If every salary is to be revised and every pension brought again on the table at every annual meeting, not only must the waste of time be so great as to deter good men from taking part in these meetings, but a feeling of uncertainty as to the good faith of the institution and the trustworthiness of the decisions of a properly constituted board must quickly become apparent among the paid officers of the hospital. It appears that it has been the custom at the Radcliffe Infirmary to allot £200 a year for the payment of a chaplain. When it was proposed that the late chaplain should retire it was arranged that a pension of £100 per annum should be paid to him by the Infirmary, and that so long as this went on only the remaining £100 per annum should be expended out of the Infirmary funds upon the salary of his successor. The Bishop of Oxford was appointed chaplain, and it was left to him to appoint a deputy who should do the work, and a supplementary fund was raised by which £30 a year was added to the amount paid as stipend by the Infirmary.

Now it would seem sufficiently obvious to most people that if the salary were to be discussed again, so must the whole question. The reverend gentleman who proposed that the salary should be increased by £50 out of the Infirmary funds seemed to imagine that the first £100 was safe and unrecallable, and that nothing could be lost by having a shot at an increase. We fancy, however, that had the matter come to a serious discussion he might have found reason to recall his early school days, and to remember what Æsop taught him about a dog and a leg of mutton. A very little common sense ought to have shown how fragile a structure was last year's arrangement, and how readily it might fall the wrong way if it were tampered with. As we pointed out at the time, the proceedings of the meeting at which it was concocted were by no means altogether open and above board. The governors were asked to buy "a pig in a poke," and they did so; which was going pretty far. They can hardly now be asked to pay a higher price than they then gave without being given the option of breaking off the bargain altogether—an eventuality which does not seem to have entered into the calculation of those who asked for the salary to be increased.

